# Unravelling the impact of climate change on honey bees: An ensemble modelling approach to predict shifts in habitat suitability in Queensland, Australia

**DOI:** 10.1002/ece3.11300

**Published:** 2024-04-18

**Authors:** Sarasie Tennakoon, Armando Apan, Tek Maraseni

**Affiliations:** ^1^ School of Surveying and Built Environment University of Southern Queensland Toowoomba Queensland Australia; ^2^ Institute of Environmental Science and Meteorology University of the Philippines Diliman Quezon City Philippines; ^3^ Institute for Life Sciences and the Environment University of Southern Queensland Toowoomba Queensland Australia; ^4^ Chinese Academy of Sciences Northwest Institute of Eco‐Environment and Resources Lanzhou China

**Keywords:** *Apis mellifera*, biomod2, climate change, ensemble modelling, honey bees, species distribution modelling

## Abstract

Honey bees play a vital role in providing essential ecosystem services and contributing to global agriculture. However, the potential effect of climate change on honey bee distribution is still not well understood. This study aims to identify the most influential bioclimatic and environmental variables, assess their impact on honey bee distribution, and predict future distribution. An ensemble modelling approach using the biomod2 package in R was employed to develop three models: a climate‐only model, an environment‐only model, and a combined climate and environment model. By utilising bioclimatic data (radiation of the wettest and driest quarters and temperature seasonality) from 1990 to 2009, combined with observed honey bee presence and pseudo absence data, this model predicted suitable locations for honey bee apiaries for two future time spans: 2020–2039 and 2060–2079. The climate‐only model exhibited a true skill statistic (TSS) value of 0.85, underscoring the pivotal role of radiation and temperature seasonality in shaping honey bee distribution. The environment‐only model, incorporating proximity to floral resources, foliage projective cover, and elevation, demonstrated strong predictive performance, with a TSS of 0.88, emphasising the significance of environmental variables in determining habitat suitability for honey bees. The combined model had a higher TSS of 0.96, indicating that the combination of climate and environmental variables enhances the model's performance. By the 2020–2039 period, approximately 88% of highly suitable habitats for honey bees are projected to transition from their current state to become moderate (14.84%) to marginally suitable (13.46%) areas. Predictions for the 2060–2079 period reveal a concerning trend: 100% of highly suitable land transitions into moderately (0.54%), marginally (17.56%), or not suitable areas (81.9%) for honey bees. These results emphasise the critical need for targeted conservation efforts and the implementation of policies aimed at safeguarding honey bees and the vital apiary industry.

## Graphical abstract

This study uses climate data from 1990 to 2009, environmental variables, and honey bee presence and pseudo absence data to produce three models: climate‐only, environment‐only, and a combined climate and environment model, along with suitability maps. Moreover, honey bee distribution is predicted for two future time spans: 2020–2039 and 2060–2079.

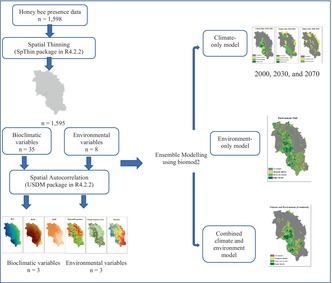

## 1 INTRODUCTION

Climate is a major factor that governs the spatial and temporal distribution of a species (Adhikari et al., 2023; Araújo et al., 2005; Pant et al., 2021). Climate change is referred to as a systematic and gradual change in average weather conditions (Weber, 2010) and these changes have serious implications on the distribution, physiology, and proliferation of a wide range of species including pollinators (Vercelli et al., 2021). The various elements of climate change, encompassing different aspects of temperature, rainfall, extreme events, carbon dioxide concentration, and ocean dynamics, are expected to impact biodiversity across all levels, ranging from individual organisms to entire biomes (Bellard et al., 2012; Parmesan et al., 2011). At the fundamental tier of biodiversity, climate change has the capacity to reduce the genetic diversity of populations through directional selection and swift migration. This, in turn, has the potential to influence the functioning and resilience of ecosystems (Botkin et al., 2007). Furthermore, the diverse impacts on populations are expected to alter the interconnected relationships within communities (Gilman et al., 2010; Walther, 2010). Essentially, the reaction of certain species to climate change may result in an indirect influence on species that rely on them. An examination of 9650 interspecific systems, encompassing pollinators and parasites, indicated that approximately 6300 species could face extinction as a consequence of the disappearance of their associated species (Koh et al., 2004). Thus, in order to survive under changing climatic conditions, any species has to either cope, adopt in situ, or shift from the current geographical locations (Maggini et al., 2011). This emphasises the importance of predicting the future distribution of a species under changing climate since such predictions can inform scientists and decision makers about future risks. In turn, this would enable the development of risk mitigation strategies to reduce the impact of climate change on biodiversity.

The pollinator species that is widely used globally to enhance agricultural production is the European honey bee or the western honey bee (*Apis mellifera*) (Hung et al., 2018; Potts et al., 2010), hereafter referred to as the honey bee. The honey bee is ranked number one as the most frequent pollinator for crops worldwide and floral species in natural habitats (Hung et al., 2018). The threat imposed by climate change on honey bees is multi‐faceted, with significant influences on diseases, parasites, predators, parasitoids, viruses, pesticide use (Cornelissen et al., 2019; Le Conte & Navajas, 2008; Varikou et al., 2020; Vercelli et al., 2021; Zawislak et al., 2019), and most importantly, the plants on which honey bees forage (Goulson et al., 2015). These issues have a huge impact on the behaviour, physiology and distribution of honey bees (Goulson et al., 2015). The future changes in honey bees with respect to the population and geographic distribution can have impact on ecology, and agriculture. Shifts in the distribution of honey bees with changing climate imply the importance of understanding the future distribution patterns for conservation purposes, preserving the ecosystem dynamics and related social systems (Bonebrake et al., 2018; Pecl et al., 2017).

The distribution of bees is hugely impacted by climate and environmental factors (Le Conte & Navajas, 2008). Specifically, their geographical presence is linked to the abundance of flowering plants, given that honey bees forage on nectar and pollen as their primary sources of food (Tennakoon et al., 2023). Honey bee foraging is significantly influenced by climatic factors, including rainfall, low temperatures, and high winds (Rowland et al., 2021). Climate conditions, encompassing temperature and precipitation patterns, further determine the availability of floral resources, ultimately affecting the foraging success of honey bees (Anderson et al., 2012; Dorji et al., 2020). Moreover, climatic conditions play a pivotal role in shaping the prevalence and propagation of diseases and parasites affecting honey bees (Giliba et al., 2020; Rowland et al., 2021; Switanek et al., 2017). Extreme temperatures have the potential to disrupt their foraging and flight capabilities (Clarke & Robert, 2018). While only a limited number of studies have attempted to model the distribution of the European honey bee (Otto et al., 2016), numerous investigations into land suitability assessment for honey bees have revealed that a diverse array of factors, primarily encompassing the availability of pollen and nectar sources, plays a pivotal role in identifying the optimal habitat locations for honey bees (Gallant et al., 2014; Smart et al., 2016).

Species distribution modelling (SDM), also known as ecological niche modelling or habitat suitability modelling, is gaining more popularity over the other tools of analysis available for ecologists to predict the distribution of species (Tikhonov et al., 2020). The aim of SDM is to provide an insight on the spatio‐temporal assembly of a species and the anticipated future distribution against the climatic and environmental changes (Guisan & Rahbek, 2011). Most importantly, SDM can be used not only for natural ecosystems but also for human managed ecosystems (Woodin et al., 2013). Throughout history, people have consistently observed associations between species distribution and the physical environment (Elith & Leathwick, 2009). Although early scientific writings leaned heavily on qualitative descriptions (Grinnell, 1904), contemporary approaches extensively employ numerical models to depict patterns and make predictions. SDMs encompass the compilation of data on species occurrences, correlating these occurrences with environmental factors, and producing maps that forecast species distributions in the past, present, or future (Pecchi et al., 2019). Most importantly, SDM can be used not only for natural ecosystems but also for human managed ecosystems (Woodin et al., 2013).

Honey bees are the most commonly used species in the beekeeping industry in Australia, making an annual contribution of $14.2 billion to the economy (Agrifutures Australia, 2022). This study models the distribution of honey bees based on bioclimatic and environmental variables and predicts their future distribution for two different time spans in the future (i.e. 2020–2039 and 2060–2079) within the context of Australia. More specifically, the objectives of this study are the following: (1) to identify the bioclimatic and environmental predictor variables that contribute the most to the distribution of honey bees, and to quantify their relative impact on honey bee distribution; (2) to assess the predictive performance of an ensemble approach in modelling the distribution of honey bees using bioclimatic and environmental variables, and (3) to investigate the potential impact of climate change on honey bee distribution under 2020–2039 (referred to as 2030) and 2060–2079 (referred to as 2070) climate conditions. This study introduces several innovations: (1) it is the first to use an ensemble approach for assessing the distribution of *Apis mellifera* in relation to bioclimatic and environmental variables; (2) it employs a relatively high‐resolution climate data (250 m); and (3) it evaluates the distribution of *Apis mellifera* under changing climate conditions, considering two future time spans.

## 2 MATERIALS AND METHODS

### 2.1 Study area

**FIGURE 1:**
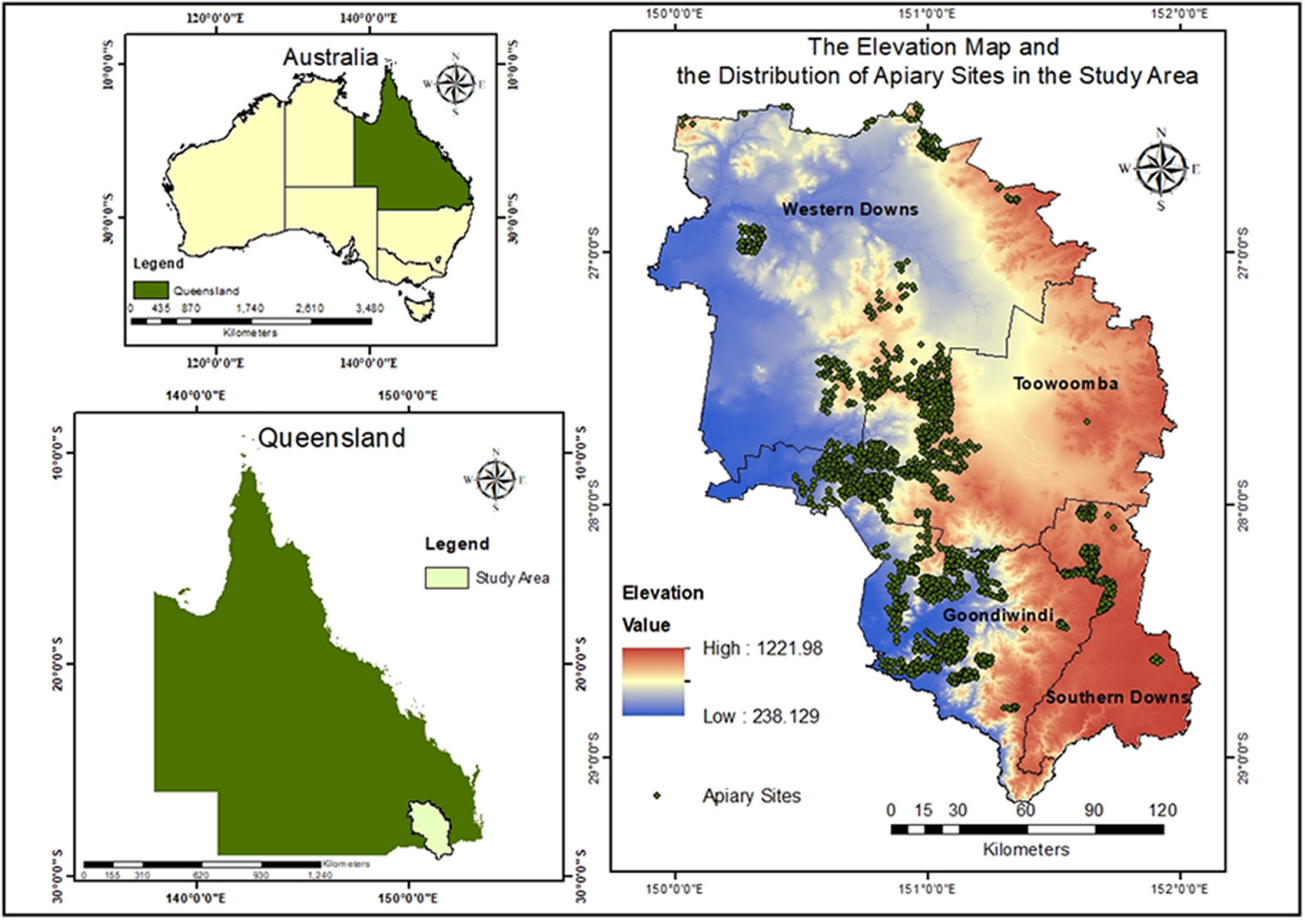
Elevation map and the apiary site locations in the study area.

As the study area, a sub‐section of Southern Queensland, Australia that covers an extent of 37,650 km^2^ encompassing the four Local Government Areas of Toowoomba, Southern Downs, Goondiwindi, and Western Downs (Figure 1) was selected. The Queensland agricultural sector contributes over $10 billion annually to the national economy (Business Queensland, 2022) and accounts for 9.7% of the country's total honey production, producing approximately 37,000 tonnes per annum (Department of Agriculture Fisheries and Forestry, 2023). Most importantly, the study area was chosen to include approximately 35% of the total managed apiary sites in Queensland's primary honey‐producing region (Queensland Spatial Catalogue, 2021), while also capturing variations in climate, topography, land cover, and land use (Queensland Spatial Catalogue, 2014).

This area exhibits significant climate variations, ranging from warm temperate conditions in Toowoomba and Southern Downs to hot arid conditions in Goondiwindi and Western Downs. For example, in Stanthorpe, Southern Downs, the mean minimum temperature during winter is 1.1°C while the mean maximum temperature in summer can reach 27.4°C. The mean annual rainfall in the same area is 764.2 mm. In contrast, Miles, Western Downs experiences a minimum temperature of 3.6°C during winter, with a mean maximum temperature of 33.3°C in summer. The mean annual rainfall in Miles is 643.4 mm (Australian Bureau of Statistics, 2022). Topographical features, such as slope, aspect, and elevation, also exhibit significant differences among various localities. For instance, the elevation is higher in Southern Downs and the Toowoomba region ranging from 690 to 1200 m above mean sea level, while most parts of the Western Downs and Goondiwindi are situated in comparatively lower elevated areas encompassing 200–300 m above mean sea level. The land use map in the study area primarily consists of regional ecosystems (basically composed of remnant and non‐remnant forests), rangelands and agriculture, collectively covering 98% of the total extent with roughly equal percentages. The secondary level classification system of the land use map reveals a diverse array of land use categories, spanning residential, agricultural, mining areas, and nature conservations.

### 2.2 Overview of the research methods

**FIGURE 2:**
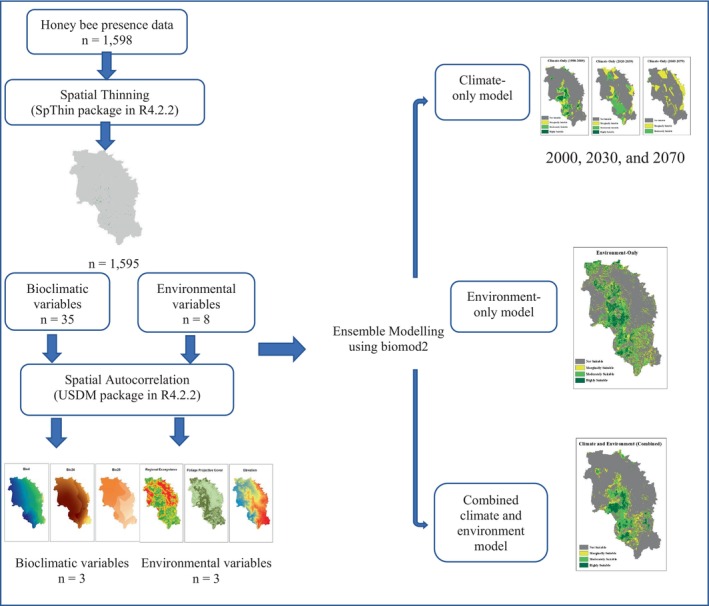
Overview of the research methods.

The overview of the research methods used in this study is shown in Figure 2. The modelling procedure of this study follows the ODMAP (overview, data, model, assessment and prediction) protocol introduced by Zurell et al. (2020). Honey bee occurrence data, along with the most influential bioclimatic and environmental variables, were utilised to develop three distinct models, a climate‐only model, an environment‐only model and a combined climate and environment model. The climate model, utilising data spanning from 1990 to 2009, was projected for the time intervals of 2020–2039 and 2060–2079.

### 2.3 Honey bee presence data

This study aimed to utilise two disparate categories of presence data, including managed apiary site locations and records of observations derived from different sources such as the Atlas of Living Australia (ALA) and the Global Biodiversity Information Facility (GBIF). The apiary site locations on public lands were retrieved from the Queensland Spatial catalogue, and the database contains 1592 records. Occurrence data from 1990 to the present year were obtained using ALA and GBIF. The study period was selected to encompass the available climate data from 1990 onwards. Only human and machine observations were included, excluding preserved specimens or museum records, as these do not accurately represent the true geographic distribution of a species (Araujo & Guisan, 2006). GBIF did not have any presence or absence records of *Apis mellifera* for the study area during the specified time, while ALA had only six records of occurrences. Thus, the bulk of the presence data was acquired from the Queensland Spatial Catalogue.

The spatial resolution of the environment and climate raster layers used in this study was 250 m × 250 m. Occurrence of multiple presence data within this resolution (within a cell) can lead to spatial sampling bias (Aiello‐Lammens et al., 2015), spatial autocorrelation (Pant et al., 2021) and overestimated measures of prediction accuracy (Veloz, 2009). Therefore, the SpThin package in R 4.2.2 was utilised to perform spatial thinning of the presence records (Aiello‐Lammens et al., 2015), with a specified buffer of 250 m as the minimum allowed distance between two occurrences. The implementation of this buffer ensured a more transparent and controlled approach, resulting in a total of 1595 records after removing only three from the initial dataset. This can be attributed to the fact that the managed apiary site locations, which serve as the primary occurrence data in this study, are established while maintaining a reasonable distance between two sites in accordance with government regulations (Biosecurity Act, 2014).

### 2.4 Bioclimatic and environmental variables

For this study, initially, eight environmental variables that impact honey bees and the apiary industry were selected based on the existing literature. These variables included regional ecosystems/flora criterion (Sarı et al., 2020), foliage projective cover (FPC), land use (Otto et al., 2016), land cover (Clermont et al., 2015), topographical features (slope, aspect, elevation), and distance to water bodies (Zoccali et al., 2017). A more comprehensive explanation of the biological relevance of these criteria to beekeepers' decision‐making have been provided in the study by Tennakoon et al. (2023). Bioclimatic variables derived from temperature and rainfall values are often used in SDM, representing annual trends, seasonality, and extremes in these climate factors. Thirty‐five bioclimatic variables covering the time periods 1990–2009, 2020–2039, and 2060–2079 at a finer scale (250 m × 250 m) were sourced from the New South Wales (NSW) and Australian Capital Territory (ACT) Regional Climate Modelling (NARCliM) database (Hutchinson & Xu, 2015).

**FIGURE 3:**
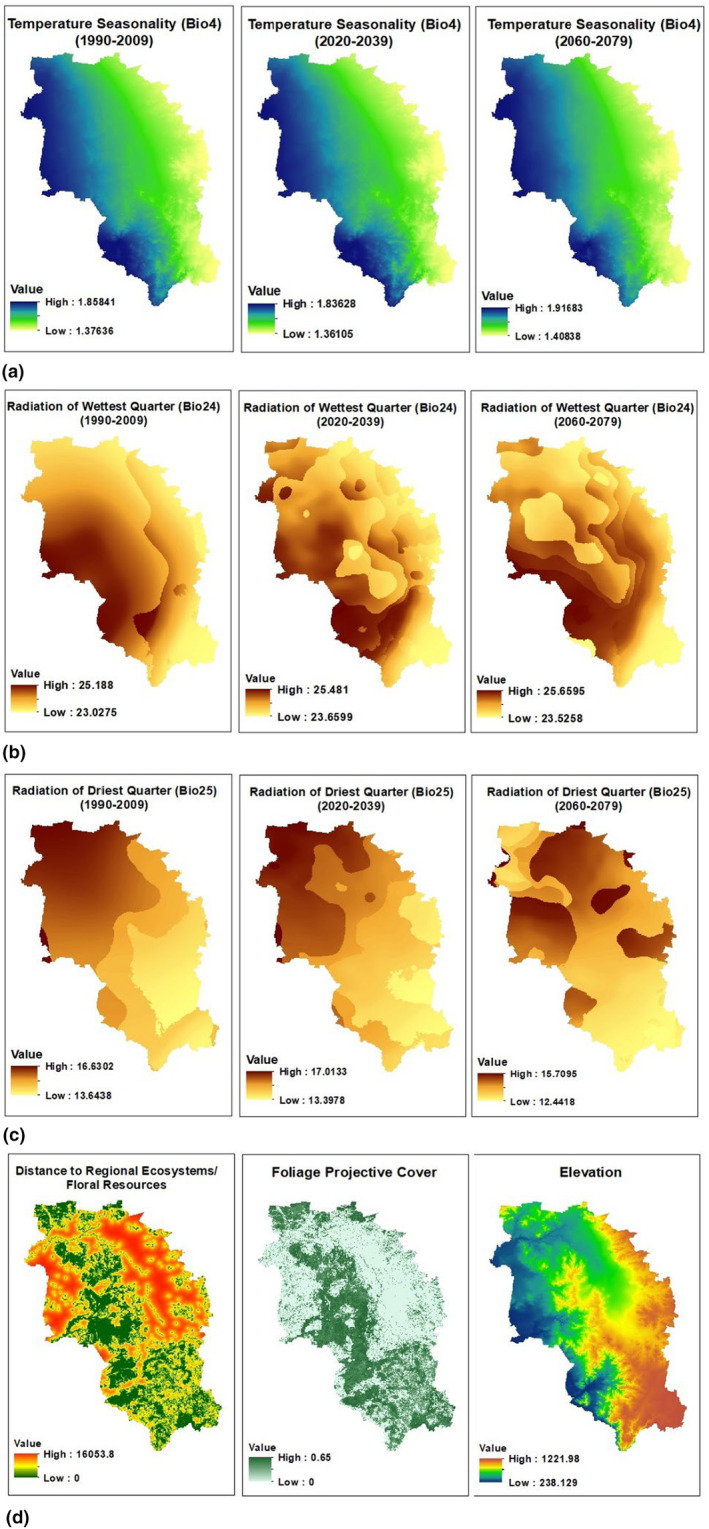
Bioclimatic and environmental variables (a) Temperature seasonality maps of 1990–2009, 2020–2039 and 2060–2079; (b) Radiation of wettest quarter (Bio24) maps of 1990–2009, 2020–2039 and 2060–2079; (c) Radiation of driest quarter (Bio25) maps of 1990–2009, 2020–2039 and 2060–2079; (d) Environmental variables (Distance to regional ecosystems (floral resources), FPC and elevation).

**FIGURE 4:**
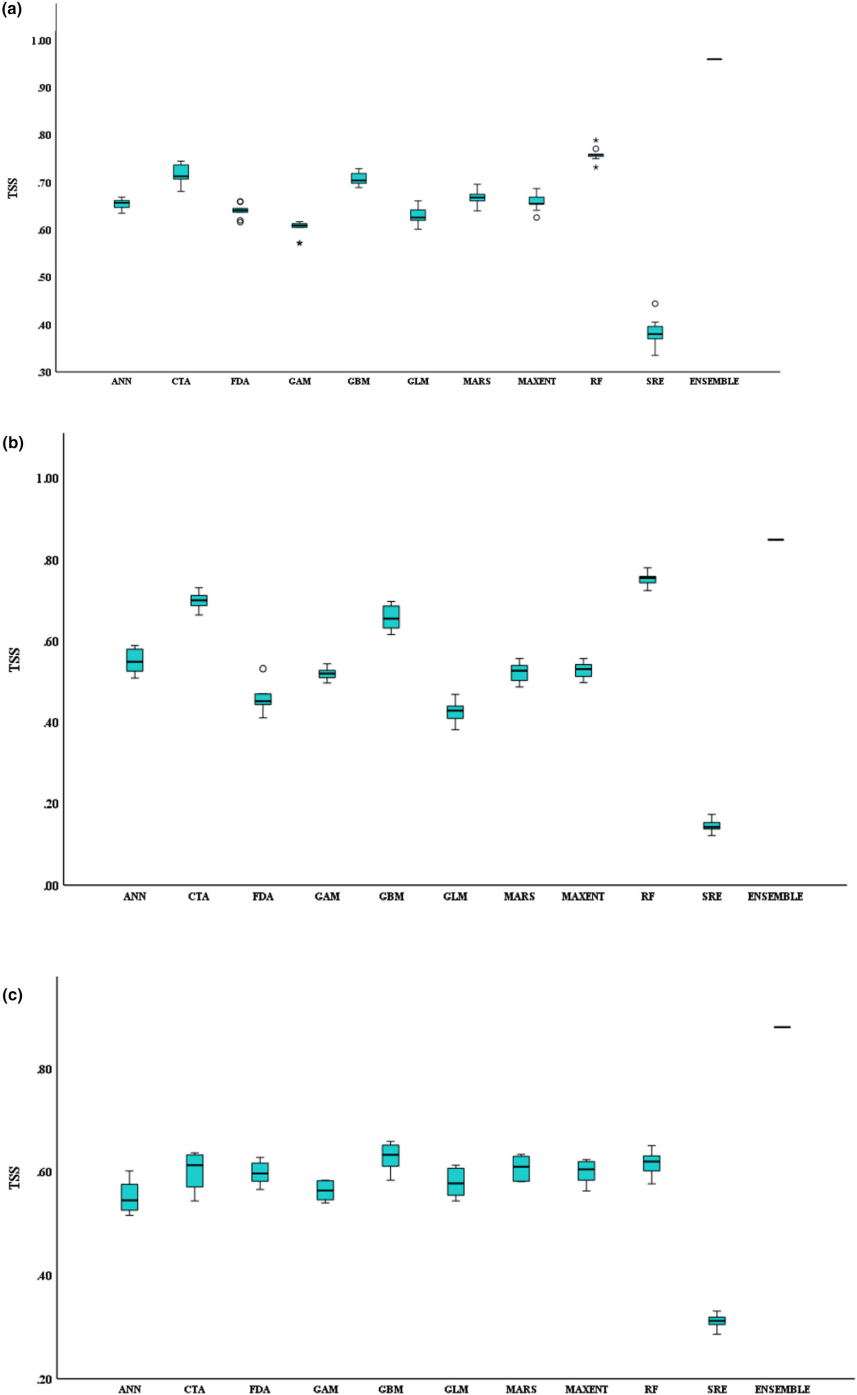
(a) TSS scores of individual algorithms and the ensemble model (climate‐only); (b) TSS scores of individual algorithms and the ensemble model (environment‐only); (c) TSS scores of individual algorithms and the ensemble model (combined).

**FIGURE 5:**
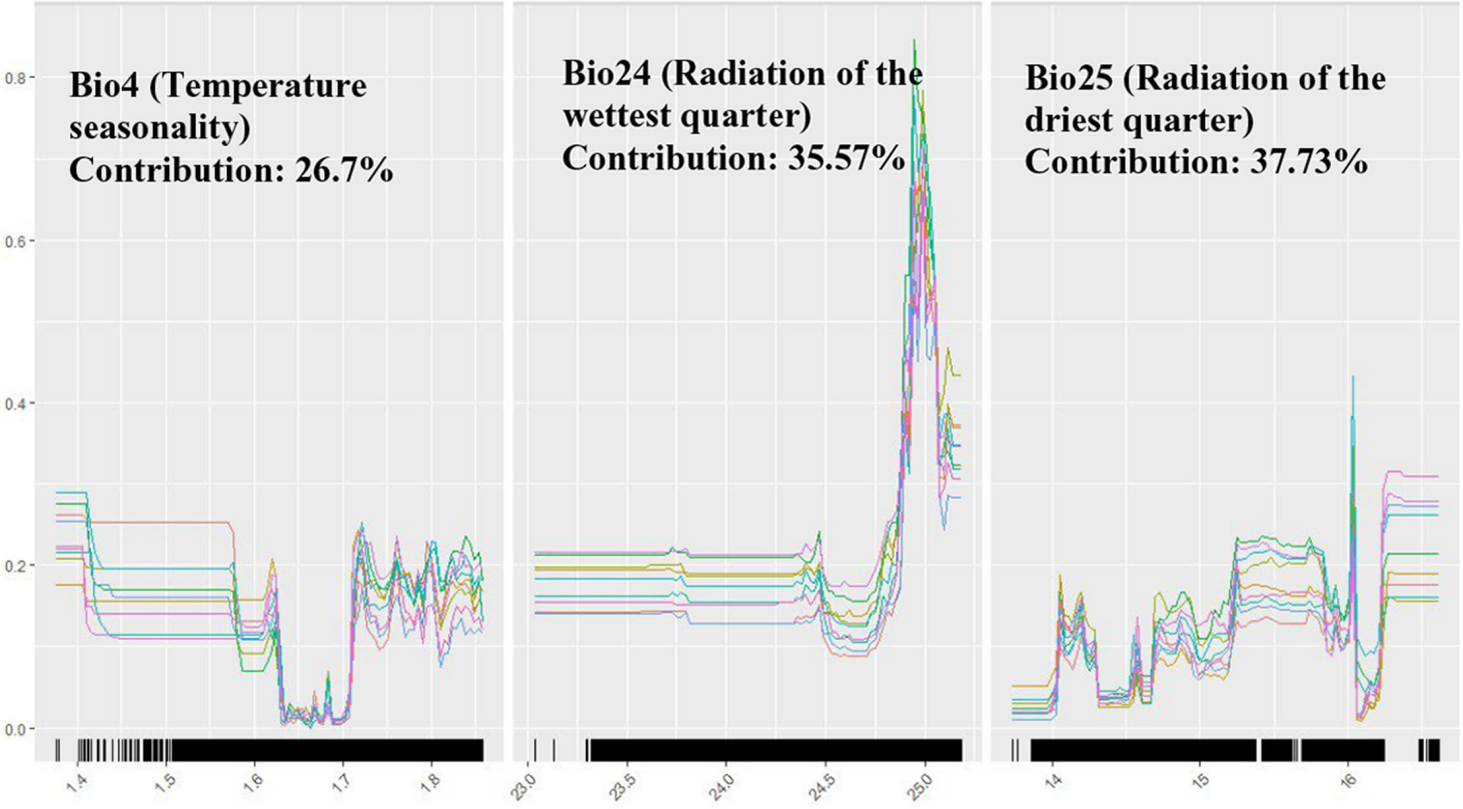
Response curves of bioclimatic variables in the climate‐only model.

**FIGURE 6:**
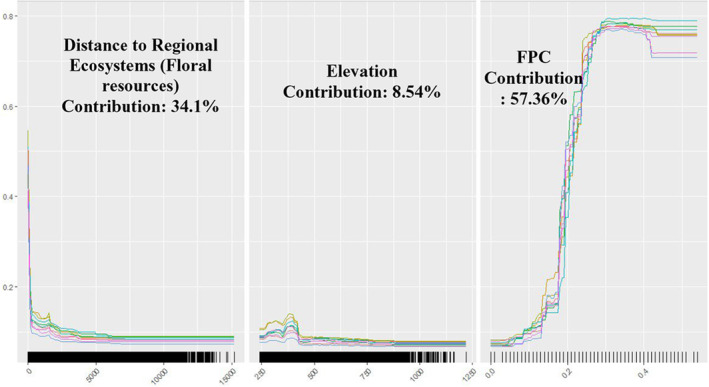
Response curves of environmental variables in the environment‐only model.

All the variables selected for modelling were tested for multicollinearity using the USDM (Uncertainty Analysis for Species Distribution Models) package (Imdadullah et al., 2016) on the R platform. Two indicators, namely the correlation coefficient and variance inflation factor (VIF), were employed as measures of multicollinearity. Principal component analysis is a powerful tool used to overcome the problem of multicollinearity (Lafi & Kaneene, 1992; Sulaiman et al., 2021). Yet, in this study, in line with the objectives of identifying the most contributing variables and quantification, principal component analysis was opted for correlation coefficient and VIF. Multicollinearity can increase uncertainty in model parameters and decrease the predictive performance of the model (De Marco & Nóbrega, 2018). Variables with a correlation coefficient greater than 0.8 and a VIF higher than 5 were excluded from further analysis, following previous studies on SDM (Diao & Wang, 2014; Fois, Bacchetta, et al., 2018; Fois, Cuena‐Lombraña, et al., 2018). All eight environmental variables were retained, while only four bioclimatic variables (i.e., Bio4, Bio15, Bio24, Bio25) remained after conducting multicollinearity testing. During the model formation process, the number of predictor variables was reduced (Breiner et al., 2015) by iteratively removing the least contributing variables to mitigate overfitting (Zeng et al., 2016). This was achieved through scrutinisation of the variable importance scores to identify those with minimal impact, and they were designated for removal. The selection of variables for exclusion was guided by their individual contributions to the overall accuracy of the model. The variables used for the final model formation, along with their sources, are listed in Table 1, whilst Figures 3, 4, 5, 6 visualise these variables. ArcMap 10.8.2 was used to create raster layers with a cell size of 250 m × 250 m and the WGS84 projection.

**TABLE 1:** Bioclimatic and environmental variables finally utilised for the ensemble modelling.

Predictor variable	Rationale	Source
Bioclimatic variables		
Bio4 (Temperature seasonality)	Temperature has a huge impact on honey bee mortality (Switanek et al., 2017), activity (Abou‐Shaara et al., 2017; Huang & Robinson, 1995), and reproduction (Rangel & Fisher, 2019)	New South Wales (NSW) and Australian Capital Territory (ACT) Regional Climate Modelling (NARCliM) (Hutchinson & Xu, 2015)
Bio24 (Radiation of wettest quarter) (Wm‐2) Bio25 (Radiation of driest quarter) (Wm‐2)	Having a significant amount of solar radiation is particularly desirable during winter because the rate at which bees leave the hive (bee egress rate) is influenced by temperature and radiation. Previous studies have observed a reduced bee egress rate when exposed to low temperatures and limited solar radiation (Clarke & Robert, 2018). Solar radiation is also associated with defensive behaviour of honey bees (Southwick & Moritz, 1987)	
Environmental variables		
Regional ecosystems (Floral resources)	Honey bees gather nectar and pollen from various flowering species, which are crucial for their survival and honey production. Hence, honey bees are present in areas where they have access to floral resources. Furthermore, when choosing a location for an apiary, it is essential to consider the availability of food sources (nectar/pollen) for honey bees. The Queensland regional ecosystems database contains information about vegetation communities in a specific bioregion. Regional ecosystems refer to vegetation communities in a bioregion that consistently correspond to specific combinations of geology, landform, and soil (Sattler & Williams, 1999). This database, therefore, serves as an excellent resource to identify the floral species suitable for honey bees in a particular ecosystem. The same methodology used to rate regional ecosystems by Tennakoon et al. (2023) was used in the present study	Regional Ecosystems Maps – Queensland Spatial Catalogue: Queensland Government (https://qldspatial.information.qld.gov.au)
Foliage projective cover (FPC)	FPC refers to the proportion of the ground surface taken up by the vertical projection of foliage (Queensland Spatial Catalogue, 2014). Foliage is an important factor related with honey bee foraging being an indicator of food sources available for honey bees and the incoming solar radiation (Specht, 1981; Steven et al., 1986)	Queensland Spatial Catalogue: Queensland Government (https://qldspatial.information.qld.gov.au)
Elevation	Elevation is closely correlated with floral resources and climatic factors that affect honey bees	GEODATA 9 Second Digital Elevation Model (DEM‐9S) Version 3 from Geoscience Australia (https://ecat.ga.gov.au) (Hutchinson et al., 2008)

### 2.5 Species distribution modelling: ensemble approach using biomod2

Biomod2 (Thuiller et al., 2016) is extensively used across different locations around the world in distribution modelling of a wide range of taxa mostly using presence only data and environment and climate factors (Hallgren et al., 2019). Biomod2 permits running 10 different modelling algorithms (Appendix S2), model calibration, evaluation, building ensembles, ensemble forecasting and visualisation of data and results (Thuiller et al., 2016).

#### 2.5.1 Appendix S2: An overview of the different modelling algorithms available in biomod2

Even though, some algorithms such as rectilinear envelope and distance‐based envelope can handle presence‐only data, most of the modelling algorithms utilise both presence and absence data. Moreover, it is proven that the presence–absence models perform better than presence‐only models (Elith et al., 2006). However, collecting absence data, particularly for mobile species, and ensuring its accuracy when compared with presence data, can be a challenging task (MacKenzie & Royle, 2005). Researchers rely on pseudo absence or background data to enhance the predictive performance of the model. For this study, 5000 pseudo‐absence points were generated using biomod2 in R 4.2.2, taking into consideration the varying number of pseudo‐absence points required for each algorithm (Barbet‐Massin et al., 2012). The decision to use 5000 pseudo‐absence points reflects the intention to establish a robust and well‐balanced dataset for training the SDM model. Equal weight was assigned to the presence and absence points, and the process of generating pseudo‐absences was repeated three times to alleviate random bias. A training dataset was used to estimate the predictive power of the model, ensuring that the training data are not spatially autocorrelated with test data (Allouche et al., 2006). In cases where independent data is unavailable for training the models, the original dataset is divided into two parts: training data and testing data. The honey bee presence and pseudo‐absence data were divided into training (80%) and testing (20%) sets, following the approach recommended by previous studies (Chapman et al., 2019; Hopkins, 2009; Laman et al., 2018; Senay & Worner, 2019; Waldock et al., 2022). In addition to the method of random splitting of data, stratified sampling (Marchetto et al., 2023), K‐fold cross‐validation, leave‐one‐out cross‐validation, temporal split, and spatial split have been employed in SDM research. The modelling process consists of a total of 90 model runs, which includes 10 modelling algorithms, three pseudo absence generation runs, and three evaluation runs. Using the ensemble modelling option available in biomod2, an ensemble species distribution model was constructed by applying multiple algorithms above a selected threshold.

### 2.6 Model evaluation

Model evaluation in biomod2 consists of an assessment of the explanatory power using a standard approach associated with each algorithm and evaluating the predictive power of the model using AUC i.e., area under the relative operating characteristic curve (ROC) (Hanley & McNeil, 1982), Cohen's Kappa (Monserud & Leemans, 1992) and the true skills statistics (TSS) (Allouche et al., 2006). AUC considers two aspects: sensitivity, which is the proportion of presences correctly predicted as presence, and specificity, which is the proportion of absences correctly predicted as absences. AUC can range from 0 to 1, with a practical range of 0.5–1. A value of 0.5 indicates a random model, while a value of 1 indicates a perfect model (Hallgren et al., 2019). Several studies on SDM have applied AUC as it eliminates the perceived subjectivity linked to threshold selection in the process. Yet the literature has strongly criticised AUC as a misleading measure of the performance of predictive distribution models (Jiménez‐Valverde, 2012; Lobo et al., 2008, 2010). The Kappa statistic evaluates the degree to which models predict occurrence at a level that exceeds what would be expected by chance (Monserud & Leemans, 1992). The Kappa statistic can have values ranging from −1 to +1. Values of 0 or below indicate random performance, while a value of +1 indicates perfect agreement (Allouche et al., 2006). The TSS considers both omission (proportion of presences identified as absences) and commission errors (proportion of absences identified as presences), and has a range of −1 to +1, where a value of +1 indicates perfect agreement, and values of zero or less indicate performance no better than random. Unlike Kappa, TSS is not influenced by prevalence. Additionally, TSS is unaffected by the size of the validation set, and two methods with equal performance will have equal TSS scores (Allouche et al., 2006).

### 2.7 Model development

In this study, three models namely the climate‐only model, the environment‐only model, and the combined climate (1990–2009) and environment model were developed. The climate‐only model was developed using the three most influential bioclimatic variables for honey bees, namely Bio4 (temperature seasonality), Bio24 (radiation of the wettest quarter), and Bio25 (radiation of the driest quarter). Only individual models with a TSS greater than 0.7 were utilised for ensemble model building. The three environmental variables with the highest contribution to the model, i.e., proximity to regional ecosystems (floral resources), foliage projective cover, and elevation were used in building the environment‐only model. Unlike the TSS values of individual algorithms pertaining to the climate‐only model, the TSS values of algorithms in environment‐only model were less than 0.7. Thus, a cut‐off TSS of 0.6 was selected when building the ensemble environment‐only model. The combined climate and environment model was developed by incorporating the environmental and bioclimatic variables from both environment‐only and climate‐only models. These variables included foliage projective cover, proximity to regional ecosystems, elevation, Bio4, Bio24, and Bio25.

### 2.8 Generation of suitability maps for current and projected climate change

Suitability maps were generated using biomod2 for each scenario under consideration, namely: climate‐only (1990–2009), environment‐only, and the combined climate and environment model. Using ensemble forecasting, suitability maps for the two future scenarios, i.e., 2020–2039 and 2060–2079 were generated. Each output map was divided into four suitability classes, based on the criterion namely: highly suitable (with a probability of occurrence exceeding 75%), moderately suitable (with a probability of occurrence ranging from 50% to 75%), marginally suitable (with a probability of occurrence between 25% and 50%), and not suitable (with a probability of occurrence less than 25%). For this manual method of reclassification, the reclassify tool in ArcMap 10.8.2 was utilised.

## 3 RESULTS

### 3.1 Model performance

#### 3.1.1 Climate‐only model

Among the algorithms used in ensemble modelling, random forest (RF) had the highest average TSS value of 0.77, followed by classification tree analysis (CTA) with a value of 0.72, while surface range envelope (SRE) had the lowest TSS of 0.27. Algorithms such as artificial neural networks (ANN), generalised additive model (GAM), generalised boosting method (GBM), generalised linear model (GLM), multivariate adaptive regression spines (MARS), and MAXENT also had average TSS values less than 0.7 (Figure 4). Consequently, these algorithms were excluded from ensemble modelling. Radiation variables including Bio24 and Bio25, had the highest contribution to the model, each accounting for 35.57% and 37.73% respectively. Bio4 or the temperature seasonality contributed to the model by 26.70%. According to the response curve pertaining to probability of honey bee occurrences and radiation in the wettest quarter, the optimum radiation for honey bees is 25 Wm^−2^. Based on the response curve for radiation in the driest quarter, honey bee occurrences display a fluctuating pattern as the radiation increases, with sudden increases and declines but an overall increasing trend. However, the optimum radiation value for honey bees in the driest quarter or winter is observed as 16 Wm^−2^. It is apparent that honey bee occurrences are limited when the temperature seasonality or Bio4 ranges between 1.6 and 1.7. Otherwise, the pattern remains relatively stable (Figure 5). The ensemble climate‐only model exhibited strong predictive performance, achieving a TSS of 0.85, an AUC of 0.98, and a Kappa value of 0.72. The same bioclimatic variables were used to project the model's predictions into the 2020–2039 (2030) and 2060–2079 (2070) periods.

#### 3.1.2 Environment‐only model

GBM had the highest average TSS value of 0.63, while RF and ANN also performed comparatively well in modelling honey bee presence data against environmental variables, achieving an average TSS of 0.62. MARS demonstrated good performance as well, with a TSS of 0.61, slightly lower than that of GBM, RF, and ANN. On the other hand, MAXENT had a TSS of 0.6. SRE, similar to the climate‐only model, demonstrated the least predictive performance, achieving a TSS of 0.31 (Figure 4). FPC made the most significant contribution to the model, accounting for 57.36% of the total. Following was the distance to regional ecosystem or floral resources, which contributed 34.10%. The elevation had the least impact on the model, contributing only 8.54% to the model. According to the response curve for regional ecosystems, honey bee occurrences are optimised near the regional ecosystems with floral resources for honey bees. There was a sharp decline as the distance from regional ecosystems increases. The probability of honey bee occurrences increases with FPC and reaches its peak when FPC is 0.3. Beyond this point, the curve remains stable. Elevation displays a rather constant pattern but with a spike between 375 and 425 m (Figure 6). The ensemble environmental‐only model showed strong predictive performance similar to the climate‐only model, with a TSS of 0.88, an AUC of 0.98, and a Kappa value of 0.75.

#### 3.1.3 Combined climate and environment model

Just like in the climate‐only model, in the combined model, RF was the best‐performing algorithm with an average TSS score of 0.76. CTA and GBM also had average TSS values of 0.72 and 0.71, respectively. Comparable to the other two models, SRE displayed the lowest TSS of 0.38 (Figure 4). A TSS threshold of above 0.7 was chosen to construct the combined model. The greatest contribution to the model came from Bio24 (radiation in wettest quarter), accounting for 27.74%, followed by distance to regional ecosystems (floral resources) and foliage projective cover (FPC) with approximately equal percentages of 21.25 and 21.63 correspondingly. The contribution of Bio25 (radiation in driest quarter) accounted for 18.36%. On the other hand, Bio4 (temperature seasonality) and elevation, which were the least influential variables in the model, had values of 5.44% and 5.58%, respectively. As per the combined model, the predictor variables behave similarly to the individual models. The combined climate and environment model demonstrated strong predictive performance, with a high TSS score of 0.96, a near‐perfect ROC score of 0.99, and a Kappa value of 0.92. Therefore, it is evident that the prediction of honey bee occurrences can be enhanced by using both environmental and climate variables together in the same model as the predictor variables.

### 3.2 Land suitability for honey bees

Based on the climate‐only model, the area classified as highly suitable experiences a drastic decline of approximately 88% from the initial period of 2000 to the projected period of 2030 (Table 2). These areas were relegated into the moderately suitable and marginally suitable categories. Furthermore, this highly suitable area is completely lost from 2030 to 2070. Conversely, the moderately suitable area demonstrates an increase of 58% from 2000 to 2030 but experiences a significant loss of 96% from 2030 to 2070, suggesting a potential future loss of areas with high and moderate suitability. The area classified as marginally suitable has more than doubled between 2030 and 2070. However, there is a decrease in the area classified as not suitable from 2000 to 2030 by 9%, followed by an increase of 15% from 2030 to 2070. It is worth noting that the not suitable area is significantly large when compared to other suitability categories.

**TABLE 2:** Suitable area (km^2^) for honey bees based on climate‐only (2000, 2030, 2070), environment‐only, and combined environment and climate model.

Classification	Climate‐only						Environment‐only		Combined (environment and climate)	
	1990–2009 (2000)		2020–2039 (2030)		2060–2079 (2070)					
	Area (km^2^)	Percent (%)	Area (km^2^)	Percent (%)	Area (km^2^)	Percent (%)	Area (km^2^)	Percent (%)	Area (km^2^)	Percent (%)
Highly suitable	1832	4.86	227	0.6	0	0	3748	9.96	2056	5.47
Moderately suitable	3546	9.42	5588	14.84	207	0.54	5159	13.72	3476	9.24
Marginally suitable	2936	7.80	5068	13.46	6611	17.56	4486	11.93	2980	7.92
Not suitable	29,336	77.92	26,767	71.10	30,832	81.90	24,220	64.39	29,101	77.37
Total	37,650	100	37,650	100	37,650	100	37,613	100	37,613	100

**FIGURE 7:**
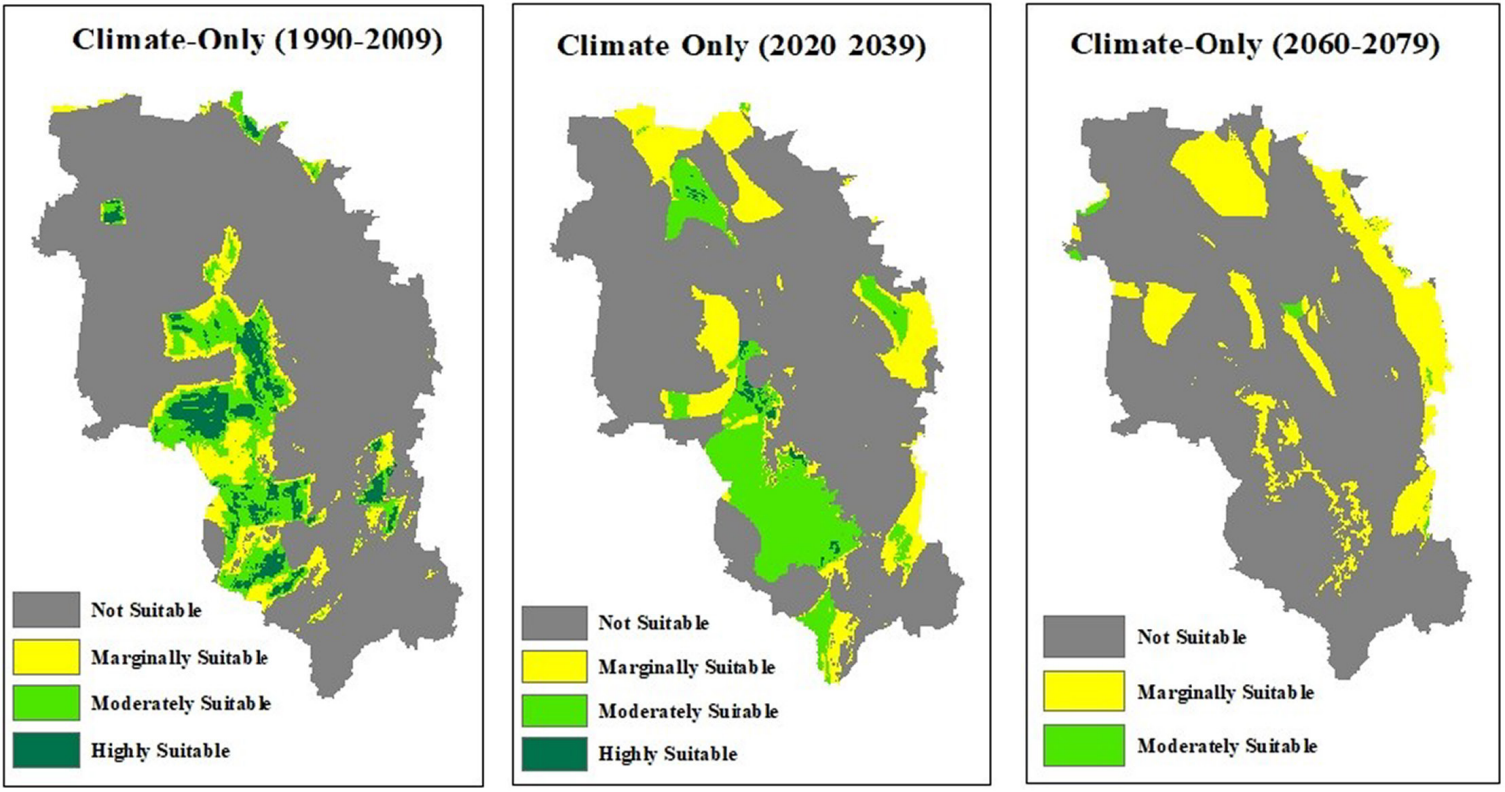
Suitability maps for honey bees: Climate‐only scenario in 1990–2009, 2020–2039 and 2060–2079.

**FIGURE 8:**
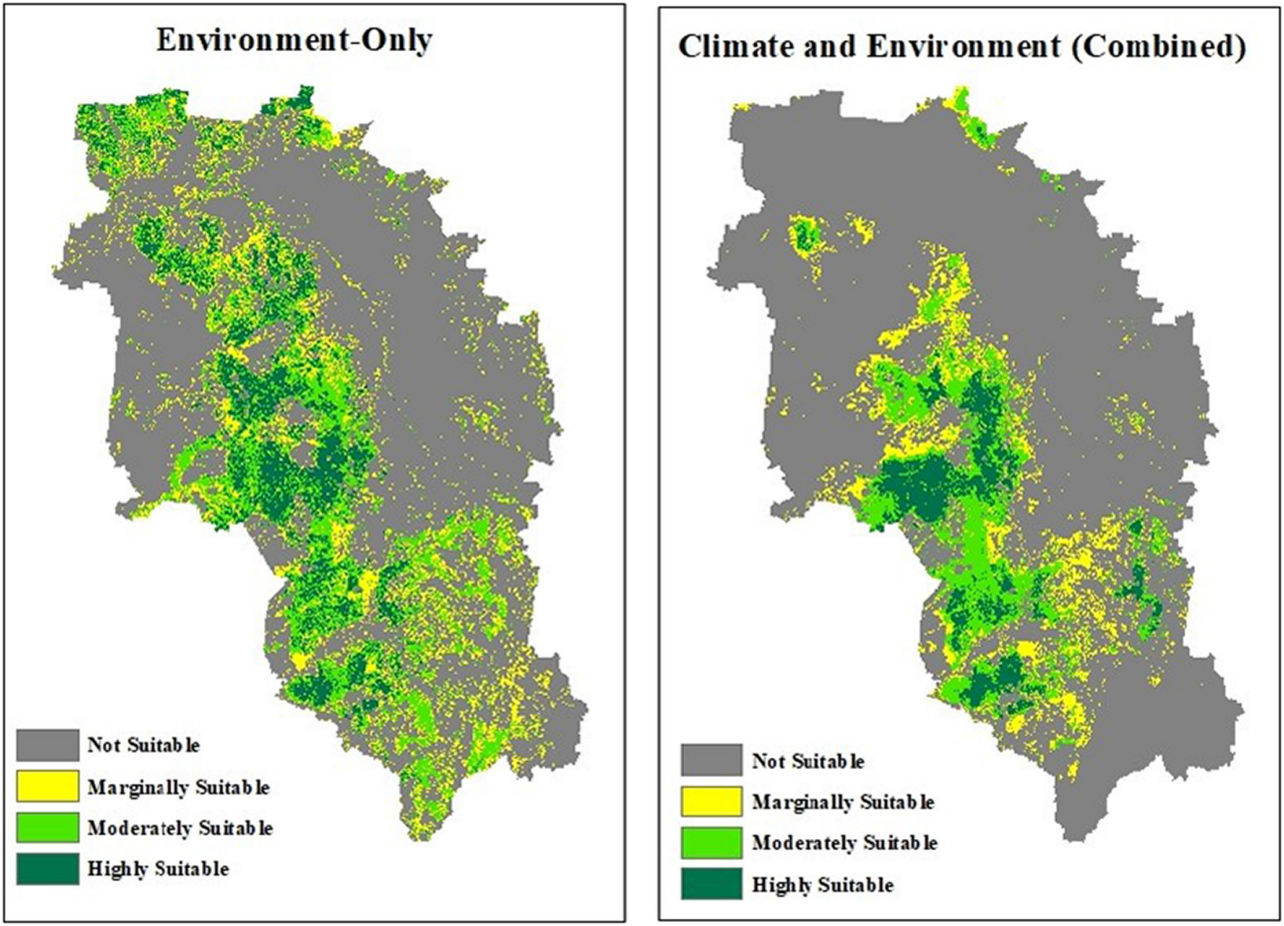
Suitability maps for honey bee habitat: environment‐only and combined (Climate and Environment) scenarios.

In the context of the environment‐only model, the highly and moderately suitable area, which accounts for 24% of the total extent, surpasses the same area pertaining to any other climate scenario or the combined model in size. The climate‐only model for 2030 indicates a significantly lower value of only 15% for the highly and moderately suitable area, making it the second‐largest value. On the other hand, the marginally and not suitable area resulted by environment‐only model is comparatively smaller, representing 76% of the total extent. In comparison, this value increases to approximately 85% for the 2000 and 2030 climate scenarios as well as the combined model, with a remarkably high value of 99% projected for 2070. This indicates that the study area offers more favourable environmental conditions for honey bees compared to suitability based on climatic factors alone. When compared with the combined climate and environment model, the highly and moderately suitable areas are larger in the environment‐only model, while they are smaller in the climate‐only model (Figures 7 and 8).

The number of honey bee occurrences was recorded for each suitability class using the sample tool in ArcMap. The results show that the highest number of honey bee locations, accounting for approximately 72%, was found in the highly suitable class of the current climate‐only model (2000). However, this number experiences a significant decline over the timeline from 2000 to 2070, indicating a complete loss of highly suitable areas by 2070. In contrast to the area distribution within each suitability class between the environment‐only and combined models, the number of occurrences in the highly suitable area is higher in the combined model compared to the environment‐only model. Only eight honey bee occurrences were found in the not suitable area under the climate‐only scenario. However, this number increases by approximately 89% in 2070, indicating a significant loss of highly and moderately suitable areas for honey bees in terms of climate (Table 3).

**TABLE 3:** Number of honey bee occurrences by suitability class under each modelling scenario.

Classification	Climate‐only						Environment‐only		Combined (environment and climate)	
	1990–2009 (2000)		2020–2039 (2030)		2060–2079 (2070)					
	Number	Percent (%)	Number	Percent (%)	Number	Percent (%)	Number	Percent (%)	Number	Percent (%)
Highly suitable	1140	71.93	18	1.13	0	0	745	47.03	1054	66.54
Moderately suitable	395	24.92	547	4.51	1	0.001	618	39.02	413	26.07
Marginally suitable	42	2.65	206	13.00	173	10.92	170	10.73	79	4.99
Not suitable	8	0.5	814	51.36	1411	89.02	51	3.22	38	2.40
Total	1585	100	1585	100	1585	100	1584	100	1584	100

## 4 DISCUSSION

### 4.1 Predictive performance of the models and contribution of predictor variables

The TSS, AUC, and KAPPA values of the climate‐only ensemble model were 0.85, 0.98, and 0.72, respectively, indicating that the model was robust with strong predictive power. A TSS value greater than 0.8 and AUC value higher than 0.9 indicate an excellent model (Hosmer & Lemeshow, 2000; Lin & Chiu, 2018; Pittman & Brown, 2011), while a Kappa value of 0.61 to 0.8 exhibits substantial performance (Landis & Koch, 1977; Viera & Garrett, 2005), which is the case in the current scenario. Anyway, TSS is argued to be a more reliable measure in assessing the predictive performance of species distribution models. This is because TSS possesses all the advantages of Kappa while not being affected by the prevalence of a species, unlike Kappa (Allouche et al., 2006). Among the 10 modelling algorithms utilised, RF had the highest TSS value, which agrees with the outcome of previous studies where an ensemble approach is employed to model species distribution (Marmion, Parviainen, et al., 2009; Williams et al., 2009). SRE was excluded from further analysis due to its poor performance in predicting the honey bee distribution which was indicated by a TSS value of 0.27. SRE is not commonly used in recent literature due its lower performance when compared with other modelling algorithms used in SDM (Pecchi et al., 2019). CTA which was included in ensemble model of the current study due to a TSS value greater than 0.7, is gaining more popularity in SDM and is argued to provide a favourable trade‐off, offering comparable accuracy to GLM or GAM (Thuiller et al., 2003).

On the other hand, the environment‐only model, incorporating predictor variables, proximity to regional ecosystems, FPC, and elevation, exhibited a high predictive performance with a TSS of 0.88, an AUC of 0.98, and a kappa value of 0.75. Unlike the ensemble model, the TSS values of individual algorithms in environment‐only model were less than 0.7. Therefore, a threshold value of 0.6 was chosen, while for the other two models the threshold was set as 0.7. If the presence data and the algorithms remain the same and only the predictor variables are different, the smaller TSS values in the environment‐only model can be attributed to the lower effectiveness of the environmental variables in explaining the underlying patterns and relationships within the data when compared to the climatic variables. This is further confirmed by the fact that, according to the climate‐only model, a higher number of honey bee occurrences align with the highly and moderately suitable classes when compared to the environment‐only model. Nonetheless, the combined environment and climate model also displayed a robust predictive power with a TSS 0.96 of ROC 0.99 of and a Kappa value of 0.92. Thus, it is evident that combining climate and environmental predictor variables in a model enhances the predictive performance. Moreover, to enhance the predictive performance of the models while mitigating problems associated with SDM, such as overfitting, several precautions were taken. These included rarefying the presence data, selection of a minimum number of predictor variables, and performing cross‐validation using 80% of the data for model calibration and 20% for validation (Pant et al., 2021).

According to the climate‐only model, the most influential variables in the model were Bio24 and Bio25 which represent the radiation of wettest quarter and radiation of driest quarter, correspondingly. Bio4 (temperature seasonality) also exhibits a significant influence on honey bee distribution. This is consistent with previous findings that solar radiation and temperature are the two most detrimental climatic factors that contribute to bee activity (Clarke & Robert, 2018). Moreover, it has been proven that bee abundance is highest in the areas with high solar insolation (Orr et al., 2021). Compared to the significance of the other two criteria, namely proximity to regional ecosystems and FPC, in constructing the environment‐only model, the contribution of elevation is minimal (8.54%). Nonetheless, elevation remains a crucial factor determining honey bee activity and has been extensively utilised in literature concerning land suitability analysis for apiary sites (Fazel & Abdul, 2012; Maris et al., 2008; Sarı et al., 2020; Zoccali et al., 2017). Furthermore, it was evident that elevation holds greater importance when compared to other topographic factors such as slope and aspect. The outcome further confirms the fact that access to floral resources is a prime criterion to be considered when locating a commercial apiary site (Tennakoon et al., 2023).

### 4.2 Response of the spatial distribution of honey bees to climate change in Australia

By the 2020–2039 period, approximately 88% of highly suitable habitats for honey bees are projected to transition from their current state to become moderate to marginally suitable areas. Due to climate change, this transformation is predicted to result in a complete change of highly suitable habitats to different categories by the years 2060–2079. However, there was a contrasting trend observed in the moderately suitable area, which showed a notable increase of 58% from 1990–2009 to 2020–2039. This increase can be attributed to favourable changes in climatic factors, such as a slight decline in temperature seasonality (Bio4) and an increase in radiation during the wettest (Bio24) and driest quarters (Bio25). However, the projection from 2020–2039 to 2060–2079 revealed a significant decline in the moderately suitable area, primarily due to an increase in temperature seasonality and a drastic reduction in radiation during the driest quarter. This indicates the potential challenges that lie ahead for honey bee habitats due to changing climate. Additionally, while there is a temporary decrease in the not suitable area by the 2020–2039 period, it subsequently increases by 2060–2079, highlighting the persistence of adverse climatic conditions for honey bees. Among the three scenarios, the environment‐only model exhibited the largest extent of highly and moderately suitable areas for honey bees, accounting for 24% of the total extent. This emphasises that the environmental factors in the study area are more favourable for honey bees than the climatic factors. The combined climate and environment model revealed a decrease of approximately 9% in this value, highlighting the limitations imposed by climate factors on habitat suitability.

By 2020–2039, new moderately suitable areas have emerged in all four regions, while most of the highly suitable areas have transitioned into moderately suitable or marginally suitable lands. During the period from 2030 to 2070, a discernible westward shift can be observed in the distribution of marginally suitable areas, whereas only scattered patches of moderately suitable areas are found in Toowoomba, Western Downs and Southern Downs. Over time, the regions of Goondiwindi that were once highly and moderately suitable are predicted to transition into areas classified as marginally and not suitable.

In the suitability map produced by ensemble modelling for the 1990–2009 period, 97% of honey bee occurrence records were found within the highly suitable and moderately suitable areas. This high correspondence between the model predictions and actual occurrences further validates the accuracy of the model. However, a significant decline is observed in future projections, with the occurrence records dropping to zero by 2060–2079. Remarkably, by the same period, a substantial majority, comprising 89% of the current occurrences, will be classified as not suitable, indicating a concerning shift in habitat suitability for honey bees. Regional ecosystems with floral species suitable for honey bees are mainly confined to the eastern and southern parts of the study area, encompassing areas such as Goondiwindi, Western Downs, and Southern Downs. With the changing climate, it is predicted that the habitat suitability for honey bees will shift towards the western parts, where there are fewer favourable regional ecosystems available. This implies the vulnerability of the apiary industry, particularly in the study area, which covers a significant portion of the honey‐producing region in Queensland.

### 4.3 Limitations of the present study and recommendations

Species Distribution Modelling (SDM) can be applied on both natural and managed ecosystems. This study aimed to assess the impact of climate change on both managed and feral honey bee colonies, yet a limitation encountered was the insufficient availability of honey bee occurrence records that can be derived from reliable sources. The honey bee presence data mainly consists of managed apiary site locations. While these apiary sites are presumed to capture the natural landscape attributes suitable for honey bees, it will be interesting to model honey bee distribution using other “natural” locations for the presence data.

While the most common approach in SDM studies is the use of a single data split, it has the drawback of potentially introducing bias to parameter estimation (Araujo et al., 2005). To address this concern, various resampling methods can be employed, including random subsampling, K‐fold cross‐validation, Jackknife (leave‐one‐out), and bootstrapping (Hastie et al., 2009). It is also advisable to explore the utilisation of similarity metrics such as Jaccard and Sørensen indices, or F‐measure indices (Leroy et al., 2018), as potential alternatives to address the limitations associated with AUC, TSS, and KAPPA in predictive distribution models.

Pesticides have a detrimental effect on honey bees, and their habitat suitability (Krupke et al., 2012; Tome et al., 2020; Williams et al., 2015; Zhu et al., 2014). In this study, the assessment of suitable locations did not consider the exposure to pesticides, which is recognised as a limitation. Therefore, it is recommended to incorporate pesticide exposure as a factor when determining suitable locations for honey bees. Furthermore, this study overlooks the aspect of habitat connectivity between suitable habitats for honey bees. It is suggested to include an analysis of the land use to assess the proximity and potential barriers among habitats. Integration of habitat connectivity measures into honey bee species distribution modelling, will provide insights into how the arrangement and accessibility of suitable habitats influence honey bee populations. This information will contribute to more accurate predictions of honey bee distribution and assist in identifying priority areas for conservation and management efforts. Furthermore, this study did not take into consideration the land use changes when predicting future habitat suitability for honey bees. Therefore, it is worthwhile to combine anticipated land use changes with the projected future maps to obtain more accurate results. It is also advisable to explore the utilisation of similarity metrics such as Jaccard and Sørensen indices, or F‐measure indices (Leroy et al., 2018), as potential alternatives to address the limitations associated with AUC, TSS, and KAPPA in predictive distribution models.

## 5 CONCLUSION

In this study, an ensemble modelling approach was employed for developing three models to examine the distribution of honey bees based on various predictor variables. These models include the climate‐only model, the environment‐only model, and the combined climate and environment model. The climate‐only model utilised the most dominant climatic factors that impact honey bee suitability such as radiation in the wettest and driest quarters, as well as temperature seasonality. On the other hand, the environment‐only model incorporated the environmental variables that primarily influence honey bee habitat suitability such as proximity to regional ecosystems, foliage projective cover, and elevation. To capture the collective influence of climate and environmental factors, the combined model was developed by integrating the variables used in both the climate‐only and environment‐only models. Using the climate‐only model, three suitability maps were projected for the time periods 1990–2009, 2020–2039, and 2060–2079. All three models demonstrated strong predictive performances with TSS values greater than 0.8. Under the 2020–2039 scenario, it is projected that 88% of the highly suitable land will transition to moderately suitable (14.84%), marginally suitable (13.46%), and not suitable (71.10%) areas, leaving only a 0.6% of the land as highly suitable. By the period of 2060–2079, the highly suitable area will undergo a complete transformation, transitioning entirely into other classes: moderately suitable (0.54%), marginally suitable (17.56%), and unsuitable (81.9%). This predicted loss of suitable habitats, particularly in terms of climate suitability, highlights the vulnerability of honey bees for climate change. Thus, this decline is anticipated to have significant impacts on natural ecosystems and commercial apiary management, which is a crucial contributor to the national economy.

The results of this study reveal a significant decline in the suitable area for honey bees under changing climate conditions. Therefore, this study stresses the importance of mitigating the impacts of climate change on honey bee habitats. Accordingly, investigating potential adaptation strategies for honey bee management in the face of climate change is crucial. Such strategies may include exploration of supplementary food sources for honey bees, selective breeding, innovative hive management techniques, and landscape planning to enhance honey bee resilience and minimise the negative impacts of changing climatic conditions. Additionally, engaging stakeholders, including beekeepers, farmers, and relevant government authorities, in addressing the challenges posed by climate change on honey bee distribution is essential. Evaluating the effectiveness of current policies and offering recommendations for promoting sustainable honey bee management and conservation efforts are key avenues for further exploration. These potential extensions would provide valuable insights into the complex interactions among climate change, environmental factors, and honey bee distribution. They would enhance our comprehensive understanding of land suitability for honey bees and contribute to the development of targeted conservation and management strategies.

## AUTHOR CONTRIBUTIONS

**Sarasie Tennakoon:** Conceptualization (equal); data curation (lead); formal analysis (lead); methodology (equal); software (lead); validation (equal); visualization (lead); writing – original draft (lead); writing – review and editing (equal). **Armando Apan:** Conceptualization (equal); formal analysis (supporting); methodology (equal); software (supporting); supervision (lead); validation (equal); visualization (supporting); writing – original draft (supporting); writing – review and editing (lead). **Tek Maraseni:** Conceptualization (equal); formal analysis (supporting); methodology (equal); supervision (supporting); validation (supporting); writing – review and editing (equal).

## FUNDING INFORMATION

Sarasie Tennakoon was funded by the University of Southern Queensland through Research Training Program Stipend Scholarship.

## CONFLICT OF INTEREST STATEMENT

The authors declare no conflicts of interest.

### OPEN RESEARCH BADGES

This article has earned Open Data and Open Materials badges. Data and materials are available at https://doi.org/10.5061/dryad.vdncjsz16.

## Supporting information


Appendices S1‐S4

## Data Availability

Location records and predictor variables used in model development can be accessed on DOI: https://datadryad.org/stash/dataset/doi:10.5061/dryad.vdncjsz16.
