# Supplementary material for: Unravelling the impact of climate change on honey bees: An ensemble modelling approach to predict shifts in habitat suitability in Queensland, Australia
Source: Ecol Evol. 2024 Apr 18;14(4):e11300. doi: 10.1002/ece3.11300 (PMC11024685; doi:10.1002/ece3.11300)
Supplement: Supplementary file 1 — Appendices S1‐S4 [file ECE3-14-e11300-s001.docx]

**Appendices for**

**Unravelling the Impact of Climate Change on Honey Bees: An Ensemble Modelling Approach to Predict Shifts in Habitat Suitability in Queensland, Australia**

Appendix S1: Thirty-five bioclimatic variables used for multicollinearity testing (Sourced from the New South Wales (NSW) and Australian Capital Territory (ACT) Regional Climate Modelling (NARCliM) database) (Hutchinson & Xu, 2015)

| **Variable Number** | **Variable** | **Minimum temp (°C)** | **Maximum temp (°C)** | **Rainfall (mm month-1)** | **Radiation (W m-2d-1)** | **Pan evaporation (mm d-1)** |
| --- | --- | --- | --- | --- | --- | --- |
| Bio01 | Annual mean temperature (°C) | × | × |  |  |  |
| Bio02 | Mean diurnal temperature range (mean(period max-min)) (°C) | × | × |  |  |  |
| Bio03 | Isothermality (Bio02 ÷ Bio07) | × | × |  |  |  |
| Bio04 | Temperature seasonality (C of V) | × | × |  |  |  |
| Bio05 | Max temperature of warmest week (°C) |  | × |  |  |  |
| Bio06 | Min temperature of coldest week (°C) | × |  |  |  |  |
| Bio07 | Temperature annual range (Bio05-Bio06) (°C) | × | × |  |  |  |
| Bio08 | Mean temperature of wettest quarter (°C) | × | × | × |  |  |
| Bio09 | Mean temperature of driest quarter (°C) | × | × | × |  |  |
| Bio10 | Mean temperature of warmest quarter (°C) | × | × |  |  |  |
| Bio11 | Mean temperature of coldest quarter (°C) | × | × |  |  |  |
| Bio12 | Annual precipitation (mm) |  |  | × |  |  |
| Bio13 | Precipitation of wettest week (mm) |  |  | × |  |  |
| Bio14 | Precipitation of driest week (mm) |  |  | × |  |  |
| Bio15 | Precipitation seasonality (C of V) |  |  | × |  |  |
| Bio16 | Precipitation of wettest quarter (mm) |  |  | × |  |  |
| Bio17 | Precipitation of driest quarter (mm) |  |  | × |  |  |
| Bio18 | Precipitation of warmest quarter (mm) | × | × | × |  |  |
| Bio19 | Precipitation of coldest quarter (mm) | × | × | × |  |  |
| Bio20 | Annual mean radiation (W m-2) |  |  |  | × |  |
| Bio21 | Highest weekly radiation (W m-2) |  |  |  | × |  |
| Bio22 | Lowest weekly radiation (W m-2 |  |  |  | × |  |
| Bio23 | Radiation seasonality (C of V) |  |  |  | × |  |
| Bio24 | Radiation of wettest quarter (W m-2) |  |  | × | × |  |
| Bio25 | Radiation of driest quarter (W m-2) |  |  | × | × |  |
| Bio26 | Radiation of warmest quarter (W m-2) | × | × |  | × |  |
| Bio27 | Radiation of coldest quarter (W m-2) | × | × |  | × |  |
| Bio28 | Annual mean moisture index |  |  | × |  | × |
| Bio29 | Highest weekly moisture index |  |  | × |  | × |
| Bio30 | Lowest weekly moisture index |  |  | × |  | × |
| Bio31 | Moisture index seasonality (C of V) |  |  | × |  | × |
| Bio32 | Mean moisture index of wettest quarter |  |  | × |  | × |
| Bio33 | Mean moisture index of driest quarter |  |  | × |  | × |
| Bio34 | Mean moisture index of warmest quarter | × | × | × |  | × |
| Bio35 | Mean moisture index of coldest quarter | × | × | × |  | × |

Appendix S2: An overview of the different modelling algorithms available in Biomod2

| Model | Overview |
| --- | --- |
| Artificial Neural Networks (ANN) | ANNs are non-linear models with several parameters (Thuiller et al., 2009) and are based on the function of the human brain (Lek & Guégan, 1999). This is an effective rule-based, machine learning algorithm gaining more popularity in SDM (Marmion, Luoto, et al., 2009). |
| Classification Tree Analysis (CTA) | CTA being an alternative machine learning algorithm to regression techniques uses a tree-based analysis system (Franklin, 2002; Venables & Ripley, 2013). This offers the benefit of capturing non-additive behavior and intricate interactions. Nonetheless, CTA tends to generate excessively intricate models, which can result in misleading interpretations (Breiman, 2017). |
| Generalized Additive Model (GAM) | GAM is a non-parametric extension to GLM (Hastie & Tibshirani, 1987). GAMs are better suited for more complex non-linear relationships between species and predictor variables that cannot be addressed by GLM (Yee & Mitchell, 1991). |
| Generalized Boosting Method (GBM) | GBM, a machine learning algorithm, exhibits high efficiency in data fitting, possess non-parametric characteristics, and leverages the strengths of various contemporary statistical techniques (Ridgeway, 1999). |
| Generalized Linear Model (GLM) | GLMs are mathematical expansions of linear models (McCullagh, 2019). GLMs can accommodate non-linear relationships and various statistical distributions that characterize spatial data and are closely connected to conventional techniques employed in linear modelling (Marmion, Luoto, et al., 2009). |
| Multivariate Adaptive Regression Spines (MARS) | MARS is an extension to linear regression models and important when there are large number of explanatory variables with low-order interactions (Thuiller et al., 2009). |
|  |  |
|  |  |
| Flexible Discriminant Analysis (FDA) | FDA is a classification method and important in performing classification among multiple groups (Hastie et al., 1994). |
| MAXENT.Phillips.2 | This is a specific implementation of the MaxEnt algorithm with additional features and improvements. Maxent is a machine learning algorithm that offers a precise mathematical framework, making it highly suitable for modelling species distributions (Phillips et al., 2006). |
|  |  |
| Random Forest (RF) | RF is capable of effectively managing correlated variables, accommodating larger datasets, processing a vast number of input variables, and handling missing data (Breiman, 2017). RF is regarded as one of the most precise algorithm in SDM (Iverson et al., 2008). |
|  |  |
| Surface Range Envelope (SRE) | Widely employed in SDM, yet has shortcomings such as the inability to achieve the same level of performance as certain alternative modelling techniques (Elith et al., 2006). Anyway, it continues to be favoured due to its simplicity and comprehensibility (Pecchi et al., 2019). |

Appendix S3: The ODMAP protocol followed for the development of honey bee species distribution models and prediction of habitat suitability in future

| **ODMAP Section/**  **Subsection** | **ODMAP Elements** |
| --- | --- |
| **OVERVIEW** | |
| *Authorship* | - **Authors:** Sarasie Tennakoon, Armando Apan, and Tek Maraseni - **Contact e-mail:** [sarasie.tennakoon@gmail.com](mailto:sarasie.tennakoon@gmail.com), sarasie.tennakoon@usq.edu.au - **Title:** Unravelling the Impact of Climate Change on Honey Bees: An Ensemble Modelling Approach to Predict Shifts in Habitat Suitability in Queensland, Australia |
| *Model objective* | - **Objective:** Identify the most influential bioclimatic and environmental variables and quantify their relative importance on honey bee distribution.   Predict habitat suitability for honey bees in two future time-spans: 2020-2039 and 2060-2079.   - **Target outputs:** Habitat suitability maps based three climate scenarios: 1990-2009, 2020-2039 and 2060-2079, environmental variables and a combined climate and environment variables. |
| *Taxon* | European honey bee, *Apis mellifera*, Apis, Apidae, Hymenoptera, Insecta |
| *Location* | Queensland, Australia |
| *Scale of analysis* | - **Spatial extent (Lon/Lat):** 150^0^12’ – 151^0^97’ E, 27^0^77’ – 27^0^68’ S, covering an extent of 37,650 km^2^ in Southern Queensland, Australia - **Spatial Resolution:** 250 m - **Temporal extent/time period:** Honey bee occurrence data- 1990 to present; environmental data - present, and bioclimatic variables – 1990–2009, 2020–2039 and 2060–2079 - **Type of extent boundary:** Political (Local Area Boundaries) |
| *Biodiversity data overview* | - **Observation type:** Managed apiary site locations (records), human observations, machine observations - **Response/Data type:** Presence-only |
| *Type of predictors* | Bioclimatic and environmental variables |
| *Conceptual model / hypothesis* | - **Hypothesis about species-environment relationships:** Distribution of a species is in equilibrium with the environmental and climatic factors that have an influence on that species. Honey bee distribution is mainly influenced by the bioclimatic variables, radiation in wettest and driest quarters, and temperature seasonality and the environmental variables proximity to regional ecosystems (floral resources), foliage projective cover and elevation. |
| *Assumptions* | - Species’ distribution is at equilibrium with their environment - Species presence data are a representative sample of the species distribution across the study area - Pseudo absence data/background data can be treated as absence data - All the key predictor variables of the species under consideration are accounted for in the model |
| *SDM algorithms* | **Algorithms:**  Artificial Neural Network (ANN)  Classification Tree Analysis (CTA)  Flexible Discriminant Analysis (FDA)  Generalised Additive Model (GAM)  Generalised Boosting Model (GBM)  Generalised Linear Model (GLM)  Multiple Adaptive Regression Splines (MARS)  Maximum Entropy (MAXENT)  Random Forest (RF)  Surface Range Envelope (SRE)   - **Model complexity:** Ten modelling algorithms were used - **Model averaging:** The models with TSS>0.7 were used to develop ensemble models pertaining to climate-only and the combined climate and environment scenarios whereas the models with a TSS>0.6 were incorporated in building the environment only model |
| *Model workflow* | - Included honey bee presence data pertaining to both human managed systems and natural occurrences - The presence data were rarefied using the SpThin package in R to reduce sample bias. - Initially, 8 environmental variables and 35 bioclimatic variables were selected and tested for multicollinearity using USDM (Uncertainty Analysis for Species Distribution Models) package in R to avoid model overfitting and reduce uncertainty in model parameters. - Variables with a correlation coefficient >0.8 and variance inflation factor (VIF) >5 were excluded from further analysis. - The most influential 3 bioclimatic variables and the 3 environmental variables were retained following a stepwise removal of the least contributing variables. - Five-thousand pseudo absence data were generated, and this process was repeated for three times to avoid random bias. - Presence and absence data were divided into training (80%) and testing data (20%). - The raster layers were processed to have a cell size of 250 m×250 m and projected to WGS84 coordinate system using ArcMap 10.8.1 - The modelling process consisted of 90 model runs that included ten modelling algorithms, three pseudo absence generation runs, and three evaluation runs. - Using the ensemble modelling option available in biomod2, an ensemble species distribution model was constructed by applying multiple algorithms above a selected threshold. - Three models namely the climate-only model, the environment-only model, and the combined climate (1990–2009) and environment model were developed. - The climate-only model was developed using the three most influential bioclimatic variables for honey bees, namely Bio4 (temperature seasonality), Bio24 (radiation of the wettest quarter), and Bio25 (radiation of the driest quarter). - The three environmental variables with the highest contribution to the model i.e., proximity to regional ecosystems (floral resources), foliage projective cover, and elevation were used in building the environment-only model. - The combined climate and environment model was developed by incorporating the environmental and bioclimatic variables from both environment-only and climate-only models. These variables included foliage projective cover, proximity to regional ecosystems, elevation, bio4, bio24, and bio25. - Suitability maps were generated using biomod2 for each scenario under consideration, namely: climate-only (1990–2009), environment-only, and the combined climate and environment model. Using ensemble forecasting, suitability maps for the two future scenarios i.e., 2020–2039 and 2060–2079 were generated. |
| *Software, codes, and data* | - **Modelling platform:** biomod2 package on R (Version 4.2.2) - **Code:** Code is shared in DRYAD data repository - **Data:** Data is shared in DRYAD data repository |
| **DATA** | |
| *Biodiversity data* | - **Taxon names:** *Apis mellifera* - **Taxonomic reference system:** N/A - **Ecological level:** Species level - **Data source:**   Honey bee presence data were derived from the Queensland Spatial Catalogue and Atlas of Living Australia (time period from: 1990 to present)   - **Sampling design:** N/A - **Sample size:** 1,595 presence records collected from the study area in Southern Queensland, Australia - **Absence data:** Five-thousand pseudo-absence data were generated - **Data cleaning and filtering:** SpThin package in R was used to rarefy the presence data |
| *Data partitioning* | - The honey bee presence and pseudo-absence data were divided into training (80%) and testing (20%) sets |
| *Predictor variables* | - **Predictor variables:**  1. Bioclimatic variables — Temperature seasonality (BIO4), Radiation in wettest quarter (BIO24), and Radiation in driest quarter (BIO25) 2. Environmental variables — Proximity to regional ecosystems (floral resources), foliage projective cover, and Elevation  - **Data sources:**  1. Bioclimatic variables: New South Wales (NSW) and Australian Capital Territory (ACT) Regional Climate Modelling (NARCliM) 2. Regional ecosystems and foliage projective cover: Queensland Spatial Catalogue: Queensland Government (<https://qldspatial.information.qld.gov.au>) 3. Elevation: GEODATA 9 Second Digital Elevation Model (DEM-9S) Version 3 from Geoscience Australia (https://ecat.ga.gov.au)  - **Data processing:** The raster layers were extracted, projected, and resampled using Arcmap10.8.1 - **Spatial resolution of raw data:** 250 m, 25 m - **Projection:** WGS84 |
| **MODEL** | |
| *Variable pre-selection* | - Thirty-five bioclimatic variables and eight environmental variables were selected. - The variables with correlation coefficients >0.8 and VIF>5 were removed from further analysis. Only 4 bioclimatic variables and all 8 environmental variables were retained based on the results of multicollinearity testing. - The most influential variables were retained following a process of removing the least contributing variables. |
| *Multicollinearity* | - Multicollinearity among the predictor variables were tested using the USDM (Uncertainty Analysis for Species Distribution Models) package in R. |
| *Model settings* | - Default settings for biomod2 |
| *Model estimates* | - **Model coefficient:** TSS, ROC and KAPPA - **Variable importance:** Importance of predictor variables in the three different models were calculated |
| *Model averaging / ensembles* | - To develop climate-only and the combined model, the models with a TSS>0.7 were selected whereas to develop the environment-only model, the algorithms with TSS>0.6 were selected. |
| *Non-independence* | - No test was performed to test for non-independence of the models. |
| **ASSESSMENT** | |
| *Performance statistics* | - **Performance statistics estimated on training data:** Model performances were assessed using the TSS scores |
| *Plausibility checks* | - **Response plots:** Ecological plausibility was tested using the response curves for the predictor variables. |
| **PREDICTION** | |
| *Prediction output* | - The continuous probability maps were classified into four categories as highly suitable, moderately suitable, marginally suitable and not suitable. |
| *Uncertainty quantification* | - **Algorithmic uncertainty:** Ensemble forecasting was employed to reduce model-based uncertainty and consensus method was utilised to combine outputs of individual algorithms - **Reality check:** On-ground reality was validated against the existing locations of managed apiary sites and honey bee occurrences. |

Appendix S4: Performance of models resulting from different combinations of predictor variables

|  | TSS | ROC | KAPPA | Variable Importance |
| --- | --- | --- | --- | --- |
| Climate Only |  |  |  |  |
| Bio4 + Bio24 + Bio25 | 0.85 | 0.98 | 0.72 | Bio4 36.42%  Bio24 36.73%  Bio25 26.86% |
| Environment Only |  |  |  |  |
| RE + Elevation + Distance to roads | 0.80 | 0.95 | 0.60 | RE 40.84%  Elevation 1.9%  Distance to roads 57.29% |
| RE + Elevation + Aspect | TSS value of each algorithm < 0.6 |  |  |  |
| RE + Elevation + Slope | TSS value of each algorithm < 0.6 |  |  |  |
| RE + Elevation + Distance from trees | TSS value of each algorithm < 0.6 |  |  |  |
| RE + Elevation + Distance to water | TSS value of each algorithm < 0.6 |  |  |  |
| RE + Elevation + FPC (TSS cut off 0.6) | 0.88 | 0.98 | 0.75 | RE 34.10%  Elevation 8.54%  FPC 57.36% |
| RE + FPC (TSS cut off 0.6) | 0.80 | 0.96 | 0.64 | RE 25.82%  FPC 74.18% |
| Climate + Environment |  |  |  |  |
| RE + FPC + Bio4 + Bio24 + Bio25 | 0.93 | 0.99 | 0.89 | RE 19.90%  FPC 21.42%  Bio4 7.19%  Bio24 32.34%  Bio25 19.15% |
| RE + FPC + Elevation+ Bio4 + Bio24 + Bio25 | 0.92 | 0.99 | 0.87 | RE 16.76%  FPC 24.10%  Elevation 5.57%  Bio4 5.01%  Bio24 29.63%  Bio25 18.93% |
